# Relationship between Hydration Status and Muscle Catabolism in the Aged Population: A Cross-Sectional Study

**DOI:** 10.3390/nu15224718

**Published:** 2023-11-08

**Authors:** Mateu Serra-Prat, Isabel Lorenzo, Jessica Martínez, Elisabet Palomera, Eulogio Pleguezuelos, Pau Ferrer

**Affiliations:** 1 Research Unit, Consorci Sanitari del Maresme, 08304 Mataró, Barcelona, Spain; epalomera@csdm.cat; 2 Centro de Investigación Biomédica en Red de Enfermedades Hepáticas y Digestivas (CIBEREHD), Instituto de Salud Carlos III, 28222 Majadahonda, Madrid, Spain; 3 Information Management Unit, Consorci Sanitari del Maresme, 08304 Mataró, Barcelona, Spain; ilorenzo@csdm.cat; 4 Dietetics and Nutritional Unit, Consorci Sanitari del Maresme, 08304 Mataró, Barcelona, Spain; jmartinezro@csdm.cat; 5 Rehabilitation Department, Hospital of Mataró, Consorci Sanitari del Maresme, 08304 Mataró, Barcelona, Spain; epleguezuelos@csdm.cat; 6 Tecnocampus Foundation, 08302 Mataró, Barcelona, Spain; pauferrerr@tecnocampus.cat

**Keywords:** dehydration, plasma osmolarity, muscle catabolism, 3-methyl-histidine, sarcopenia, aged

## Abstract

Background: The physiopathology of sarcopenia is still not completely understood. Aim: To assess the relationship between dehydration and skeletal muscle catabolism, muscle mass, and sarcopenia in an aged population. Methods: Observational cross-sectional study of community-dwelling subjects aged 70 years and older. Dehydration was assessed by plasma osmolarity; bioimpedance analysis (BIA) was used to assess body composition and water content; sarcopenia was established according to the EWGSOP-2 criteria; and 3-methyl-histidine (3MH) was used as an indicator of muscle catabolism. Results: 190 participants were recruited (77.4 years; 51.6% women). In total, 22.6% and 20.5% presented plasma osmolarity of 295–300 mOsm/L and >300 mOsm/L, respectively. Age was correlated with plasma osmolarity (r_s_ = 0.439; *p* < 0.001). Plasma osmolarity was correlated with 3MH (r_s_ = 0.360; *p* < 0.001) and showed an effect on 3MH levels, with an adjusted (by age, sex, and number of medications) beta of 0.283 (*p* < 0.001). BIA water content indicators showed no correlation with 3MH. Lower in sarcopenic compared to non-sarcopenic subjects were the intracellular water percentage (60.3 vs. 61.2%; *p* = 0.004) and intracellular water/free-fat mass ratio (44.3 vs. 45.0; *p* = 0.004). Conclusions: Dehydration is a highly prevalent clinical condition in aged populations, increases with age, and is associated with muscle catabolism but not sarcopenia.

## 1. Introduction

Sarcopenia is defined as age-related loss of muscle mass (MM) and strength [1]. It greatly affects quality of life, as it favours frailty, falls, and fractures [2,3], and is a risk factor for functional decline and disability [4]. These consequences of sarcopenia highlight the need to better understand its causes and to invest in its prevention. Muscle is a dynamic tissue with constant turnover. After the age of 50, there is a loss of MM of approximately 1–2% per year [5] due to a negative metabolic balance between muscle anabolism (synthesis) and catabolism (breakdown). Some mechanisms have been suggested to explain changes in MM with age, most related to a diminished capacity of the muscle to synthesize proteins due to a lack of physical exercise, poor protein ingestion, decreased anabolic hormones (insulin, testosterone, growth hormone, and insulin-like growth factor-I), anabolic resistance, muscle denervation, or mitochondrial dysfunction [6,7]. Other mechanisms refer to factors related to increased muscle protein breakdown, such as chronic inflammation with catabolic effects [8]. Despite this, the physiopathology of sarcopenia is still not completely known and further research is needed to obtain a global understanding of its causes.

It is known that body water content, i.e., the percentage of total body water (TBW) with respect to body weight, decreases with age, mainly due to a relative increase in fat mass (FM) compared to fat-free mass (FFM) [9]. However, chronic low-grade dehydration has also been reported for advanced ages, i.e., decreased water content in the FFM [10]. Indeed, it is known that aged populations are at increased risk of dehydration [11], with severe consequences for health [12]. The fact that inadequate water intake in the aged population has been associated with sarcopenia [13,14] seems logical, as muscle is the main reservoir of body water. Moreover, in aged populations, reduced intracellular water (ICW), whether measured as extracellular water (ECW)/ICW, ICW/FFM, or ICW/TBW ratios, has been associated with poor muscle strength and functional capacity, slowed gait speed, and an increased risk of frailty [15,16,17,18,19]. We hypothesize that dehydration and water depletion in the FFM may have a catabolic effect on muscle metabolism, favouring muscle breakdown, loss of MM, and sarcopenia. The aim of this study was to assess the relationship between dehydration and skeletal muscle catabolism, MM, and sarcopenia in an aged population.

## 2. Materials and Methods

### 2.1. Study Design and Population

We conducted an observational cross-sectional study of community-dwelling subjects aged 70 years and older. Inhabitants ascribed to 3 primary care centres in the Maresme region (Barcelona, Spain) were randomly pre-selected from the primary care census database and invited by telephone to a visit with their primary care physician, who informed them about the study, checked eligibility criteria, and if they agreed to participate, obtained their signed consent. Excluded were subjects with active malignancy, neuromuscular disease, bilateral knee or hip prostheses, dementia, serious mental illness, life expectancy of less than 6 months, and in palliative care or institutionalized. Recruitment took place between January and March 2020 (when it was interrupted because of the COVID-19 pandemic) in a first sampling process and, to increase sample size and improve statistical power, from April to June 2023 in a second sampling process (the initial analysis was close but did not reach statistical significance). The local ethics committee approved the study protocol (codes CEIm CSdM 65/19 and CEIm CSdM 07/23).

### 2.2. Data Collection

A fasting blood sample was obtained from 8–9 a.m., and plasma osmolarity was used as the main indicator of hydration status. Plasma osmolarity was analyzed using the Micro-osmometer Osmo1 (Advanced Instruments). Measurements were conducted by freezing point depression osmometry, based on the principle that each mole of the dissolved solute decreases the freezing point of a liquid by 1.86 °C. Results were converted to mOsm/L H_2_O. Incipient dehydration was defined as plasma osmolarity > 295 and ≤300 mOsm/L, and severe dehydration as plasma osmolarity >300 mOsm/L. Body composition was assessed by bioelectrical impedance analysis (BIA), which provides estimates of FM, FFM, and MM (in kg and as a percentage of body weight), TBW (in litres and as a percentage of body weight), and ECW and ICW (in litres and as a percentage of TBW). We used the InBody s10 multifrequency BIA device in the following standard conditions: no intense exercise in the previous 24 h, no alcohol consumption in the previous 8 h, strict fasting in the previous 2 h, and a toilet visit prior to the evaluation. Used as indicators of water content were the percentage of TBW (%TBW) with respect to body weight, the percentage of ICW with respect to TBW (%ICW), and the TBW/FFM and ICW/FFM ratios. These parameters were categorized by the 20th percentile (p20) cut-off point (for each sex separately), with participants with values below this cut-off point considered to have relatively low water content and at risk of dehydration. 

Catabolic muscle activity was measured by plasmatic levels of 3-Methyl-histidine (3MH), a semi-essential amino acid that is released in the breakdown of the myosin and actin muscle proteins. It is formed by translational modifications of certain histidine residues of the polypeptide chain. 3MH reflects muscle protein breakdown activity because it is not subject to any metabolic pathway other than protein catabolism and is excreted in the urine without being reused. Under normal conditions, levels of 3MH are proportional to MM, although there is intra-individual variability of 10–20% due to other factors such as hormonal status, degree of physical fitness, recent intense exercise, injuries, and endocrine or neuromuscular diseases [20]. Theoretically, as levels of 3MH are proportional to MM, 3MH/MM and 3MH/creatinine (3MH/Cr) ratios were used as adjusted indicators of muscle catabolic activity. 3MH is a muscle protein turnover biomarker known for more than 50 years [21], which has recently reawakened interest [22,23]. Its excretion is increased in muscle-wasting diseases [24] and, more recently, has been related to frailty [25]. Interleukin-6, C-reactive protein, GH, IGF-1 cortisol and copeptin were used as secondary indicators of catabolism. 

Sarcopenia was defined, according to the definition and criteria of the European Working Group on Sarcopenia in Older People 2nd revision (EWGSOP-2)) [1], by the presence of low muscle strength (hand grip <27 kg in men and <16 kg in women using the handheld JAMAR dynamometer) and low muscle quantity (appendicular skeletal MM (ASM)/height^2^ < 7.0 kg/m^2^ in men and <5.5 kg/m^2^ in women according to BIA). 

### 2.3. Statistical Analysis

For sample size estimation, the correlation between plasma osmolarity (as an indicator of hydration status) and 3MH was the main analysis considered. For an alpha risk of 0.05 and a beta risk of 0.10 in a two-sided test, 84 subjects were necessary to detect a statistically significant correlation coefficient ≥ 0.35. As all analyses were stratified by sex, we aim to recruit a minimum of 164 participants (84 men and 84 women). The GRANMO calculator was used to estimate sample size (http://www.imim.es/ofertadeserveis/software-public/granmo, accessed on 25 September 2023). Continuous variables were described using mean and standard deviation (SD) values, and categorical variables were described using percentages. Spearman (r_s_) correlation coefficients were used to assess the relationship between 3MH and hydration status indicators as continuous variables. The Mann–Whitney U-test was used to assess the relationship between dichotomous variables and 3MH and the 3MH/MM and 3MH/Cr ratios. Normality was assessed by the Kolmogorov–Smirnov test. The chi-square or Fisher’s exact test was used to assess associations between osmolarity and 3MH categories and dehydration and sarcopenia. Simple and multiple linear regression analyses were performed to analyze and adjust the effect of osmolarity on 3MH. As body composition and muscle function differ for men and women, analyses involving BIA parameters and strength were performed separately by sex. Statistical significance was set to *p* < 0.05.

## 3. Results

### 3.1. Study Sample

Overall, 190 participants were recruited (95 in the first period and 95 in the second) with a mean (SD) age of 77.4 (4.5) years, 98 (51.6%) of whom were women. The main comorbidities were arterial hypertension (AHT) (66.7%), arthritis (58.5%), dyslipidemia (62.8%), diabetes (25.4%), peripheral arterial disease (14.8%), depression (21.7%), gastroesophageal reflux (16.4%), cancer (14.3%), ischemic heart disease (16.4%), asthma (9.0%), stroke (7.9%), chronic obstructive pulmonary disease (COPD) (11.1%), and chronic kidney disease (CKD) (20.3%). Of the study sample, 7.0% were considered frail and 4.3% had sarcopenia. Participants took a mean of 5.4 (3.5) medications.

### 3.2. Hydration and Catabolic Indicators

Of the study sample, 22.6% presented plasma osmolarity of 295–300 mOsm/L and 20.5% presented plasma osmolarity >300 mOsm/L (i.e., incipient and severe dehydration, respectively). In all, 43.1% of the sample was therefore considered to have some degree of dehydration according to the osmolarity criteria. 

Table 1 summarizes the main hydration indicators for the whole sample and is broken down by sex and age groups. There was no difference in plasma osmolarity between the sexes, but men had higher water content than women. The percentage of subjects with plasma osmolarity >295 mOsm/L was higher among those aged ≥80 years. Age was correlated with plasma osmolarity (r_s_ = 0.439; *p* < 0.001).

3MH was not associated with age and sex, nor with comorbidities other than CKD (12.2 µmol/L CKD vs. 7.9 µmol/L non-CKD; *p* = 0.001) and AHT (9.8 µmol/L AHT vs. 6.7 µmol/L non-AHT; *p* = 0.019). 3MH showed a correlation with the number of medications (r_s_ = 0.165; *p* = 0.024). The 3MH/MM ratio showed a similar pattern, i.e., not related to sex, but related to age (0.34 <80 years vs. 0.44 ≥80 years; *p* = 0.018), was also associated with CKD (0.52 CKD vs. 0.33 non-CKD; *p* = 0.002) and AHT (0.41 AHT vs. 0.28 non-AHT; *p* = 0.039), and likewise was correlated with the number of medications (r_s_ = 0.187; *p* = 0.012). The 3MH/Cr ratio was only related to dyslipidemia (11.2 dyslipidemia vs. 8.0 non-dyslipidemia; *p* = 0.039) and the number of medications (r_s_ = 0.156; *p* = 0.033).

### 3.3. Relationship between Hydration Indicators and Muscle Catabolism

**Figure 1:**
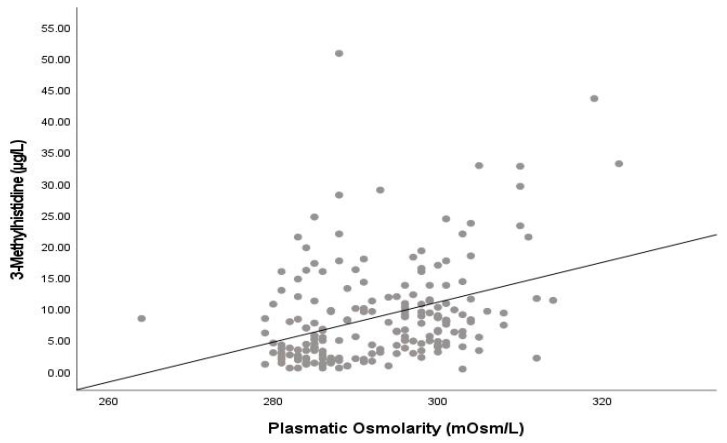
Relationship between plasma osmolarity and 3-Methyl-histidine.

Plasma osmolarity showed a positive correlation with 3MH (r_s_ = 0.360; *p* < 0.001) and showed an effect on 3MH levels with a crude beta of 0.318 (*p* < 0.001) and an adjusted (by age, sex, AHT, CKD, and number of medications) beta of 0.293 (*p* < 0.001) (see Figure 1). Plasma osmolarity was also correlated with the 3MH/MM (r_s_ = 0.390; *p* < 0.001) and 3MH/Cr (r_s_ = 0.295; *p* < 0.001) ratios. Plasma osmolarity was negatively correlated with IGF-1 (r_s_ = −0.355, *p* < 0.001) and positively correlated with copeptin (r_s_ = 0.267, *p* < 0.001) but showed no significant correlation with interleukin-6, C-reactive protein, GH, and cortisol.

Table 2 shows mean levels of 3MH, 3MH/MM, and 3MH/Cr by hydration categories. As dehydration status increased, so too did 3MH and the 3MH/MM and 3MH/Cr ratios. Of subjects with severe dehydration (plasma osmolarity > 300 mOsm/L), 12.8% presented 3MH levels above p95 (≥24.4 µmol/L), but only 2.6% of subjects presented with non-severe dehydration (*p* = 0.019) (OR: 5.40, 95% CI: 1.38–21.2). 

The BIA indicators of water content (%TBW, %ICW, TBW/FFM, and ICW/FFM) showed no correlation with 3MH or with the 3MH/MM and 3MH/Cr ratios, neither for the sample as a whole nor for men and women analyzed separately.

### 3.4. Relationship of Hydration and Water Content Indicators with MM and Sarcopenia

Severe dehydration (plasma osmolarity > 300 mOsm/L) was not related to either MM (24.3 kg severe dehydration vs. 25.1 kg non-severe dehydration; *p* = 0.450) or sarcopenia (7.7% severe dehydration vs. 3.4% non-severe dehydration: *p* = 0.367). Mean plasma osmolarity was 295 mOsm/L in sarcopenic subjects and 293 mOsm/L in non-sarcopenic subjects (*p* = 0.302). Lower in sarcopenic subjects compared to non-sarcopenic subjects were both %ICW (with respect to TBW) (60.3% vs. 61.2%; *p* = 0.004) and the ICW/FFM ratio (44.3 vs. 45.0; *p* = 0.004). However, %TBW and the TBW/FFM ratio were not significantly related to sarcopenia.

## 4. Discussion

Our results show that in the population aged ≥70 years (a) approximately 40% have some degree of dehydration (plasma osmolarity > 295 mOsm/L), and 20% have severe dehydration (plasma osmolarity > 300 mOsm/L); (b) plasma osmolarity (and risk of dehydration) is not associated with sex but increases with age; (c) 3MH is not associated with sex, slightly increases with age, and is positively correlated with plasma osmolarity in that dehydrated patients present higher 3MH serum values; (d) BIA indicators of water content are not associated with 3MH; and (e) sarcopenia is related with % ICW and ICW/FFM but not with plasma osmolarity.

Approximately 40% of our study sample aged ≥70 years were at least at risk of dehydration, and 20% were clearly dehydrated, with plasma osmolarity > 300 mOsm/L. This result agrees with those reported in other studies and in a systematic review and meta-analysis of low-intake dehydration in non-hospitalized older adults, which indicates that 24% of aged subjects present plasma osmolarity > 300 mOsm/L [26]. Those data highlight the magnitude of a health problem in the aged population that has probably not received adequate attention. We also observed that dehydration increases as age increases, corroborating previous findings indicating that this risk increases with age [11]. This would suggest the need for hydration status screening of aged populations, especially in the primary care setting, given that dehydration is a preventable clinical condition and is well known to be associated with poor health outcomes, hospitalization, and death [12,27,28,29]. Further research is needed to better understand drinking behaviour and causes of dehydration and to assess the effectiveness of drinking interventions in aged populations. No significant differences in plasma osmolarity were observed between men and women, which suggests that both sexes are at the same risk of dehydration. Note that sex differences in the BIA indicators in our study were due to sex differences in body composition (as men have higher FFM values) and not to differing dehydration risks. 

We observed that plasma osmolarity was correlated with serum levels of 3MH and that dehydrated aged subjects presented higher 3MH levels than hydrated subjects. This would suggest that dehydration may favour muscle breakdown and MM loss in the aged population. Muscle catabolism is a complex process influenced by several factors, one of which could be dehydration. Dehydration could lead to muscle catabolism in different ways. First, dehydration may reduce the efficiency of nutrient and oxygen transport to muscle cells; it may also reduce glycogen storage in muscles, so they rely more on protein as an energy source [30]. Second, dehydration stimulates arginine vasopressin (AVP) and stress hormone production and increases cortisol levels, with well-known catabolic effects [31]. We observed a positive correlation between plasma osmolarity and levels of copeptin, a surrogate of AVP, but observed no correlation with cortisol. Third, dehydration causes fatigue and muscle disuse, so muscles are not adequately stimulated, thus closing a vicious circle. Finally, dehydration, depending on severity and duration, can potentially lead to mitochondrial damage or impaired mitochondrial function due to reduced oxygen supply, increased oxidative stress (reactive oxygen species (ROS) production), disruption of energy production (because of an imbalance in ions and molecules), and, if dehydration persists over time, chronic inflammation [32]. Dehydration alone is unlikely to cause significant muscle catabolism if persistence over time is brief and is not accompanied by other factors such as physical inactivity or a chronic lack of calorie and protein intake. However, chronic or severe dehydration, combined with other stressors (a usual situation in aged populations), may contribute to muscle catabolism and breakdown. This situation is aggravated if anabolic stimuli are lacking, such as physical exercise or protein intake. Despite this, it cannot be ruled out that the correlation that we observed between plasma osmolarity and 3MH concentrations may be due, even in part, to a ‘concentration effect’ secondary to dehydration. We rule out the possibility that the positive correlation between osmolarity and 3MH is due to a hemo-concentration effect since we observed no significant differences in 3MH levels between participants for the main indicator of hemo-concentration, i.e., a high hematocrit count (>50% in men and >46% in women in our study). Moreover, when adjusting for the effect of osmolarity on 3MH levels according to a high hematocrit count, we observed a significant independent effect of osmolarity but no effect of hemo-concentration. Further studies are needed to deepen our knowledge of the effect of dehydration on muscle metabolism and the mechanisms by which dehydration affects MM and muscle function. The 3MH/MM ratio seems to increase with age, suggesting that muscle breakdown also increases with age, probably because of the increased prevalence of inflammatory diseases and catabolic states.

We observed that, despite the observed close relationship between plasma osmolarity and 3MH, BIA indicators of water content were not related to 3MH levels. This apparent contradiction has different possible explanations. BIA may not be an accurate method to assess hydration status, as it directly measures resistance and reactance and, based on these measures, indirectly estimates body composition and water content via closed algorithms, taking into account other data such as age, sex, weight, and height, and based on certain assumptions regarding specific populations. Furthermore, the different BIA parameters are estimated based on the same source data and on the mentioned assumptions, and so are closely related, prone to error, and not exempt from controversy. In any case, the gold standard to assess hydration status is plasma osmolarity [33], and the lack of correlation between plasma osmolarity and BIA indicators of water content may indicate that both methods assess different concepts. While osmolarity assesses hydration status, BIA parameters reflect body water content, which is not the same, as dehydrated subjects may have high water content because of edema, while well-hydrated subjects may have low water content because of low FFM or high FM. There is also the possibility of measurement error, e.g., from not having strictly respected implementation conditions for BIA assessment or because of the presence of metal in a body. However, we believe the first is a remote possibility, as BIA was always performed by trained and experienced professionals, while the use of metal prostheses, plates, or other orthopedic synthetic elements or metal devices is frequent in aged populations. 

Although we observed a close relationship between plasma osmolarity and 3MH, we did not find any association between plasma osmolarity and MM or sarcopenia. The lack of statistical significance may be due to a limited sample size for a relatively small effect. A prospective design would allow time of exposure and time from exposure to onset of MM decline to be assessed. Osmolarity is a very sensitive indicator that identifies dehydration in very early stages, even subclinically. However, for subclinical dehydration to have effects on MM and body composition, this dehydration must be maintained or aggravated over time or be accompanied by other catabolic stimuli. Moreover, although BIA parameters in our study were not related to 3MH, %ICW and the ICW/FFM ratio were associated with sarcopenia, indicating that sarcopenic patients have less cell mass and less MM.

As noted above, the main study limitations include (a) its cross-sectional design, which did not allow us to establish causal relationships, only associations between study factors; (b) a relatively small sample size, which determined a poor statistical power, especially in the multivariate and subgroup analyses; and (c) non-measurement of physical activity and protein intake as reflecting anabolic stimuli, which could influence results and could be used as adjusting co-variables.

## 5. Conclusions

In summary, our study shows that (a) dehydration is a highly prevalent clinical condition in aged populations; (b) dehydration increases with age; and (c) as dehydration severity increases, so also does muscle catabolic activity increase. However, we found no relationship between dehydration and sarcopenia or between BIA water content parameters and indicators of muscle catabolism. Several uncertainties remain regarding the effect of dehydration on muscle metabolism, so further research is needed, especially in longitudinal studies, to assess this effect and its impact on functional capacity in aged populations.

## Figures and Tables

**Table 1:** Mean (SD) hydration, water content, and catabolic indicators by sex and age.

	Sample N = 190	Women N = 98	Men N = 92	*p*	<80 Years N = 142	≥80 Years N = 48	*p*
	Hydration indicators						
Plasma osmolarity (mOsm/L)	292.9 (9.1)	292.0 (9.0)	293.8 (9.0)	0.197	291.8 (8.6)	296.0 (9.7)	0.002
Osmolarity (mOsm/L):							
≤290	86 (45.3%)	49 (50.0%)	37 (40.2%)	0.323	74 (52.1%)	12 (25.0%)	0.002
291–295	22 (11.6%)	9 (9.2%)	13 (14.1%)		17 (12.0%)	5 (10.4%)	
>295	82 (43.2%)	40 (40.8%)	42 (45.7%)		51 (35.9%)	31 (64.6%)	
	BIA water content indicators						
%TBW	46.4 (5.5)	43.9 (5.4)	49.0 (4.4)	<0.001	46.9 (5.6)	44.6 (5.0)	0.016
%ICW	61.0 (0.8)	60.8 (0.8)	61.2 (0.7)	0.003	61.1 (0.7)	60.6 (0.9)	0.002
TBW/FFM	73.6 (0.3)	73.5 (0.3)	73.8 (0.3)	<0.001	73.6 (0.3)	73.6 (0.3)	0.726
ICW/FFM	44.9 (0.6)	44.7 (0.6)	45.1 (0.5)	<0.001	45.2 (0.52)	44.6 (0.7)	0.003
	Catabolic indicators						
3MH (µmol/L)	8.7 (8.0)	8.2 (8.2)	9.3 (7.8)	0.165	8.4 (8.2)	9.7 (7.6)	0.069
3MH/MM (µmol/L/kg)	0.36 (0.4)	0.39 (0.4)	0.34 (0.3)	0.464	0.34 (0.3)	0.44 (0.4)	0.018
3MH/Cr (µmol/L/g)	10.0 (9.1)	11.0 (10.7)	8.9 (6.9)	0.376	9.8 (9.6)	10.5 (7.3)	0.148

Abbreviations: 3MH, 3-methyl-histidine; Cr, creatinine; FFM, fat-free mass; ICW, intracellular water; MM, muscle mass; TBW, total body water.

**Table 2:** Muscle catabolic activity indicators according to hydration status categories.

	Plasma Osmolarity (mOsm/L)				
	≤290 Hydrated	290–295 Dehydration Risk	295–300 Incipient Dehydration	>300 Severe Dehydration	*p*
N (%)	86 (45.3%)	22 (11.6%)	43 (22.6%)	39 (20.5%)	---
3MH	6.9 (7.8)	7.8 (6.6)	8.5 (4.4)	13.6 (10.2)	<0.001
3MH/MM	0.27 (0.3)	0.33 (0.3)	0.37 (0.2)	0.58 (0.5)	<0.001
3MH/Cr	9.0 (10.9)	9.1 (7.6)	10.1 (5.5)	12.7 (8.3)	<0.001

Abbreviations: 3MH, 3-methyl-histidine; Cr, creatinine; MM, muscle mass.

## Data Availability

The data that support the findings of this study are not openly available due to confidentiality norms but are available from the corresponding author upon reasonable request and Ethical Committee approval.
